# Effect of Obesity on Retinal Integrity in African Americans and Caucasian Americans With Relapsing Multiple Sclerosis

**DOI:** 10.3389/fneur.2021.743592

**Published:** 2021-11-24

**Authors:** Jacob Rube, Madeline Bross, Christopher Bernitsas, Melody Hackett, Fen Bao, Evanthia Bernitsas

**Affiliations:** ^1^Wayne State University School of Medicine and University Health Center, Detroit, MI, United States; ^2^Detroit Country Day High School, Beverly Hills, CA, United States; ^3^PPD Regional, Clinical Operations, Detroit, MI, United States; ^4^Wayne State University Imaging Laboratory and Sastry Foundation Imaging Initiative, Detroit, MI, United States

**Keywords:** optical coherence tomography, retinal integrity, obesity, multiple sclerosis, body mass index

## Abstract

**Objective:** To study the effect of obesity on retinal structures in African Americans (AAs) and Caucasian Americans (CAs) with relapsing-remitting multiple sclerosis (RRMS).

**Methodology:** About 136 patients with RRMS without history of optic neuritis were divided into two groups, based on body mass index (BMI): 67 obese (40 AA, 27 CA, mean BMI ± SD: 36.7 ± 5.8), and 69 non-obese (23 AA, 46 CA, mean BMI ± SD: 24.0 ± 3.1). The peripapillary retinal nerve fiber layer (pRNFL) thickness was quantified by optical coherence tomography (OCT) and was segmented into quadrant thickness: superior (S), inferior (I), temporal (T), and nasal (N). Papillomacular bundle (PMB) thickness, retinal nerve fiber layer (RNFL), ganglion cell + inner plexiform layer (GCIPL), inner nuclear (INL), outer plexiform (OPL), outer nuclear (ONL), and total macular (TMV) volumes were obtained.

**Results:** Obesity was associated with lower T thickness (58.54 ± 15.2 vs. 61.9 12.4, *p* = 0.044), higher INL (0.98 ± 0.07 vs. 0.96 ± 0.06, *p* = 0.034), and lower RNFL (0.77 ± 0.14 vs. 0.82 ± 0.12, *p* = 0.009) volumes. Obese AA had significantly thinner T (58.54 ± 15.19 vs. 61.91 ± 12.39, *p* = 0.033), N (68.94 ± 2.7 vs. 77.94 ± 3.3, *p* = 0.044), and TMV (8.15 ± 0.07 vs. 8.52 ± 0.09, *p* = 0.003), RNFL (0.74 ± 0.02 vs. 0.82 ± 0.02, *p* = 0.013), OPL (0.76 ± 0.01 vs. 0.79 ± 0.1, *p* = 0.050), ONL (1.68 ± 0.031 vs. 1.79 ± 0.038, *p* = 0.026), and GCIPL (1.78 ± 0.04 vs. 1.9 ± 0.05, *p* = 0.038) compared to obese CA. Among patients with non-obesity, the ONL was significantly lower in AA (1.78 ± 0.04 vs. 1.9 ± 0.05, *p* < 0.001).

**Conclusions:** Obesity is associated with retinal structure abnormalities in patients with RRMS. Its impact might be more prominent in AA than CA. Large longitudinal studies are needed to validate our findings.

## Introduction

Multiple sclerosis (MS) is an autoimmune condition characterized by combined inflammatory and neurodegenerative processes in the central nervous system (CNS) (1). The relapsing-remitting form of multiple sclerosis (RRMS) is defined by unpredictable and recurrent episodes of increased neuroinflammation and increased disability, which can often be detected using imaging measures (2, 3). Optical coherence tomography (OCT) is a non-invasive method for visualizing retinal morphology and quantifying the thickness and volume of its individual layers. Decreased retinal nerve fiber layer (RNFL) thickness has been observed in patients with MS utilizing OCT whether they have had optic neuritis or not (4–6). Reduced RNFL thickness has also shown correlation with disease progression in observational studies, whereas ganglion cell + inner plexiform layer (GCIPL) was found to reflect brain atrophy (7).

While the cause of MS is unknown, several risk factors are associated with developing MS. Obesity can contribute to persistent low-grade inflammation in the CNS, and once MS is diagnosed, obesity might negatively impact disease prognosis (8, 9). Several studies have demonstrated that having obesity in early life was associated with an increased risk of being diagnosed with MS in adulthood (10). Some studies have shown a higher prevalence of obesity in patients with MS; however, other studies have observed that rates of obesity are not as common in patients with MS with higher levels of disability (11). Although studies have found promising biomarkers and other immunological links, definitive findings regarding obesity and its effects on clinical progression of MS are not currently known. Given that obesity rates have nearly doubled since 1980, continuing to monitor obesity in clinical populations and its effects on comorbid diseases is becoming increasingly relevant (12).

Current literature stipulates that MS is diagnosed more frequently in the Caucasian (CA) population; however, clinical and imaging outcomes have demonstrated that African American (AA) patients tend to suffer from a more aggressive disease course and higher rates of disability measured by Expanded Disability Status Scale (EDSS) than CA (13–17). Little has been investigated to further understand the difference in MS disease progression and its biomarkers across racial groups (18–20). Previous studies have noted that minority populations, including the AA population, experience a higher risk of suffering from obesity (21–23). Furthermore, other studies have observed higher rates of obesity and higher body mass index (BMI) measurements in the AA population compared to the CA population (24, 25). This study aims to better understand the role of obesity on OCT measures in patients with RRMS and to determine the extent of its effect in patients of different racial backgrounds.

## Methods

Patients consented to an observational research study approved by the Wayne State University Institutional Review Board between March 2016 and December 2018. As a part of this study, they had weight and height measured, and an OCT scan performed in our clinic. A retrospective review was performed on all patients in our OCT database. Patients with a history of optic neuritis (ON), macular edema, cataracts, glaucoma, refractive errors of greater or equal of ±6 diopters, hypertension, diabetes, or a relapse <30 days of the OCT examination were excluded from this study. About 136 patients (272 eyes) with RRMS were identified and included in this study according to the criteria listed (Table 1). The 136 patients were separated into two cohorts: obese (BMI ≥ 30; 40 AA and 27 CA) and non-obese (BMI < 30; 23 AA and 46 CA). BMI was calculated based on weight in kilograms (kg) divided by height in meters squared (m^2^).

**Table 1:** Demographics summary of patients.

**Category**	**Sex (*n*)**	**Race (*n*)**	**Mean Age ± SD**	**Mean disease duration ± SD**	**Mean BMI ± SD**
Obese (*n* = 67)	Males: 16	AA: 40	AA: 42.1 ± 8.8	AA: 7.0 ± 6.9	AA: 36.8 ± 5.1
	Females: 51	CA: 27	CA: 45.9 ± 11.2	CA: 8.8 ± 7.6	CA: 36.7 ± 6.9
Non-obese (*n* = 69)	Males: 27	AA: 23	AA: 41.8 ± 10.6	AA: 10.5 ± 7.4	AA: 24.7 ± 2.9
	Females: 42	CA: 46	CA: 45.6 ± 10.4	CA: 10.9 ± 8.8	CA: 23.7 ± 3.2

*AA, African American; CA, Caucasian American*.

### Optical Coherence Tomography

All OCT scans were performed by an experienced technician on a single Heidelberg SPECTRALIS SD (Spectral Domain)-OCT with N-Site Analytics platform, software version 6.0 (Heidelberg Engineering, Inc. Heidelberg, Germany). In each OCT scan, the thickness of the papillomacular bundle (PMB) was measured along with the peripapillary retinal nerve fiber layer (pRNFL). The pRNFL was divided into four quadrants: superior (S), inferior (I), temporal (T), and nasal (N). To evaluate the thickness of the pRNFL, a single-line circular B-scan with radius of 3.4 mm from the center of the papilla was used, with average automatic real time (ART) of 80. The total macular volume (TMV) was measured within a 6-mm diameter circle centered on the fovea as the volume between the inner limiting membrane and the boundary of the retinal pigment epithelium. Macula scan used a 30 × 20 mm^2^ area composed of 61 B-scans with an average ART of 9. Fully automated, intra-retinal segmentation was performed to obtain the volumes of the following layers: retinal nerve fiber layer (RNFL), ganglion cell layer (GCL), inner plexiform layer (IPL), inner nuclear layer (INL), outer plexiform layer (OPL), outer nuclear layer (ONL), retinal pigment epithelium (RPE), and photoreceptor (PR). GCL and IPL volumes were added together (GCIPL) for statistical and reporting purposes to reduce automatic segmentation errors. For each patient, the average of both eyes was calculated, per layer and quadrant, for a total of 136 to be used for statistical analysis. All images were checked for quality purposes to avoid potential segmentation errors as described in the OSCAR IB criteria (26). Measurement of pRNFL and intra-retinal segmentation was conducted blinded to the race of the study participant. OCT study results were reported according to the consensus APOSTEL recommendations (27).

### Statistical Analysis

Statistical analysis was performed using SPSS v26 (IBM SPSS Statistics for Windows, version 26.0; IBM Corp., Armonk, NY, USA). A two-sample *t*-test was performed to determine the differences in age and disease duration between the obese group and the non-obese group. A chi-square test was performed to determine the differences in sex and race between the two groups. The relationships between pairs of clinical variables were analyzed using Spearman's rank correlation coefficient. We utilized the average value of both eyes for each patient. A general linear model with multivariate analysis of covariance (MANCOVA), with OCT measures as dependent variables and BMI as the independent variable, was used to compare OCT measures between obese group and non-obese group and between AA group and CA group, with age and disease duration as covariates. No adjustments for multiple comparisons were performed. A *p*-value of 0.05 or lower was considered to be statistically significant for all statistical tests.

## Results

### Study Population

**Figure 1:**
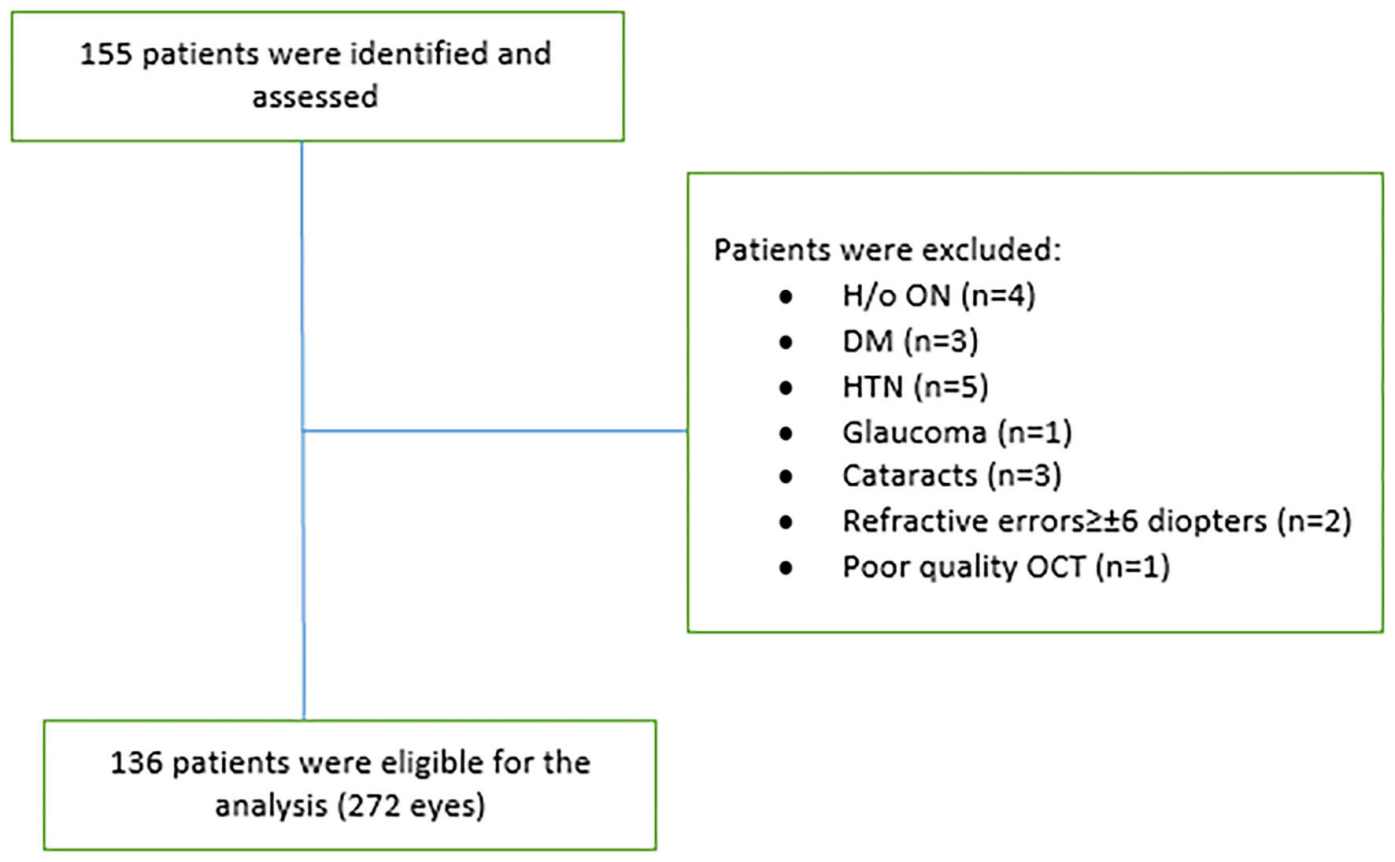
Study profile. ON, optic neuritis; DM, diabetes mellitus; HTN, hypertension; OCT, optical coherence tomography.

About 136 patients were eligible to participate in the analysis (Figure 1). There was non-significant difference between the two cohorts in age (obese: 43.6 ± 9.9; non-obese: 44.3 ± 10.5, *F* = 0.126, *p* = 0.723) or disease duration (obese: 7.7 ± 7.1; non-obese: 10.8 ± 8.3, *F* = 1.566, *p* = 0.213). There was a higher number of AA than CA in the obese group (40/67) than the non-obese group (23/69) (*p* = 0.002). Sex reached borderline significance between the two groups with a higher proportion of men in the obese group (*p* = 0.056). Disease duration is significantly correlated with BMI grouping (*r* = 0.17, *p* = 0.05) and age (*r* = 0.43, *p* = 0.00) (Table 2). One OCT scan was excluded due to poor imaging that resulted in poor data quality.

**Table 2:** Correlations of variables.

**Variables**	**Correlations**	**Age**	**BMI values**	**Disease duration**
Age	*r*	–	0.03	0.43
	*p*-value	–	0.73	0.00^*^
BMI	*r*	0.03	–	0.17
values	*p*-value	0.73	–	0.05^*^
Disease duration	*r*	0.43^*^	0.17	–
	*p*-value	0.00	0.05	–

*BMI, body mass index*.

* *Statistically significant, Spearman's rank correlation coefficient*.

### OCT in the Entire Group (AA and CA): Obese vs. Non-obese Patients With MS

**Figure 2:**
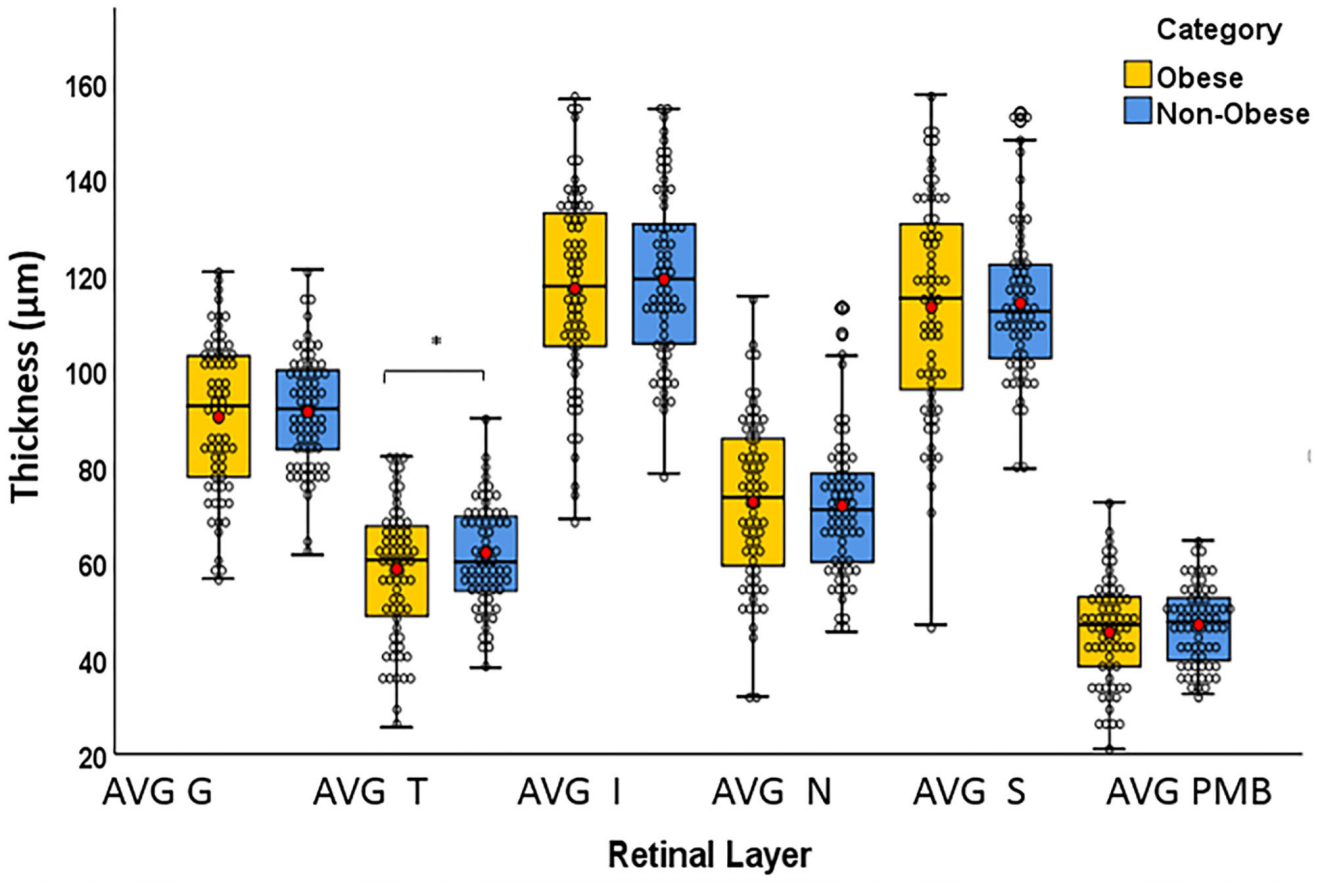
Box plots overlaid with dot plots showing retinal layer thickness by BMI category (*statistically significant). Red dots represent means. μm: micrometer. G, global; T, temporal; I, inferior; S, superior; N, nasal; PMB, papillomacular bundle. *Statistically significant.

**Figure 3:**
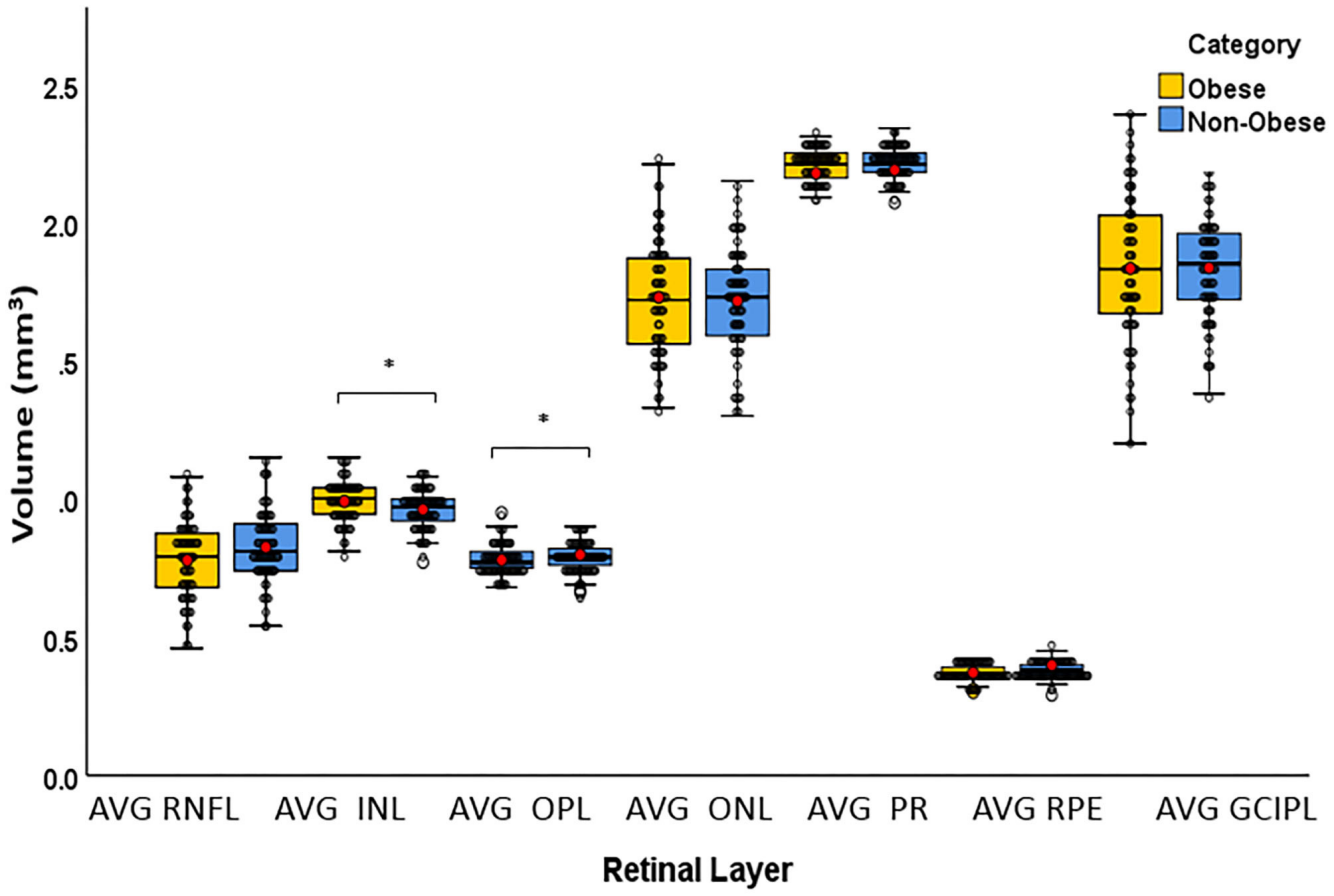
Box plots overlaid with dot plots showing retinal layer volume after intra-retinal segmentation by BMI category (*statistically significant). Red dots represent means. mm^3^, cubic millimeter; BMI, body mass index; RNFL, retinal nerve fiber layer; INL, internal nuclear layer; OPL, outer plexiform layer; ONL, outer nuclear layer; PR, photoreceptor; RPE, retinal pigment epithelium; GCIPL, ganglion cell and internal plexiform layer. *Statistically significant.

The T quadrant thickness of the pRNFL and the RNFL volume were significantly lower in the obese group than in the non-obese group, *p* = 0.044 and *p* = 0.009, respectively. The INL volume was significantly higher in the obese group than in the non-obese group (*p* = 0.034). None of the other quadrants (I, N, and S) or global (G) or the PMB were significantly different between the obese and non-obese groups. There was no volumetric significance noted in the remaining layers (TMV, OPL, ONL, PRL, RPE, and GCIPL) in the obese vs. non-obese groups (Table 3; Figures 2, 3).

**Table 3:** OCT outcome significance by category (obese vs. non-obese).

**OCT measure**	**Category**	**Mean**	**SD**	Sig. (*p-*value^*^)
AVG G (μm)	Obese	90.24	16.05	0.34
	Non-obese	91.32	11.73	
AVG T (μm)	Obese	58.54	15.2	0.04^*^
	Non-obese	61.913	12.4	
AVG I (μm)	Obese	117.03	20.24	0.34
	Non-obese	118.91	19.58	
AVG N (μm)	Obese	72.53	17.61	0.96
	Non-obese	71.73	14.87	
AVG S (μm)	Obese	113.15	22.83	0.51
	Non-obese	113.97	18.79	
AVG PMB (μm)	Obese	45.35	10.78	0.18
	Non-obese	46.94	9.93	
AVG TMV (mm^3^)	Obese	8.30	0.52	0.62
	Non-obese	8.21	0.98	
AVG RNFL (mm^3^)	Obese	0.77	0.14	0.009^*^
	Non-obese	0.82	0.12	
AVG INL (mm^3^)	Obese	0.98	0.07	0.034^*^
	Non-obese	0.96	0.06	
AVG OPL (mm^3^)	Obese	0.77	0.05	0.11
	Non-obese	0.79	0.10	
AVG ONL (mm^3^)	Obese	1.72	0.20	0.74
	Non-obese	1.71	0.19	
AVG PR (mm^3^)	Obese	2.17	0.23	0.79
	Non-obese	2.18	0.23	
AVG RPE (mm^3^)	Obese	0.45	0.70	0.53
	Non-obese	0.39	0.20	
AVG GCIPL (mm^3^)	Obese	1.81	0.26	0.26
	Non-obese	1.83	0.18	

*μm, micrometer; mm^3^, cubic millimeter; G, global; T, temporal; I, inferior; S, superior; N, nasal; PMB, papillomacular bundle; TMV, total macular volume; RNFL, retinal nerve fiber layer; INL, internal nuclear layer; OCT, optical coherence tomography; OPL, outer plexiform layer; ONL, outer nuclear layer; PR, photoreceptor; RPE, retinal pigment epithelium; GCIPL, ganglion cell and internal plexiform layer*.

* *Statistically significant, multivariate analysis of covariance (MANCOVA)*.

### OCT in the Obese Group: AA vs. CA

Within the obese group, the thicknesses of the T and N pRNFL quadrants were significantly less (*p* = 0.033 and 0.044, respectively) in the AA than in the CA group. There were no other significant findings within the pRNFL (G, I, S, or PMB) between AA and CA in the obese group. Many of the macular layers were discovered to be significantly lower in AA vs. CA in the obese group including the following: TMV (*p* = 0.003), RNFL (*p* = 0.013), OPL (*p* = 0.050), ONL (*p* = 0.026), and GCIPL (*p* = 0.038). Differences in the remaining macular layers (INL, PR, or RPE) were non-significant between AA and CA within the obese group (Table 4).

**Table 4:** BMI category breakdown of OCT measures by race (AA and CA).

**OCT measure**	**Race**	**Obese group**			**Non-obese group**		
		**Mean**	**SD**	***p*-value**	**Mean**	**SD**	***p*-value**
AVG G (μm)	AA	88.31	16.27	0.08	90.13	10.87	0.64
	CA	93.11	10.86		91.92	12.22	
AVG T (μm)	AA	55.97	13.62	0.033^*^	60.48	9.99	0.63
	CA	62.35	16.80		62.63	13.47	
AVG I (μm)	AA	114.47	21.77	0.09	118.39	16.82	0.91
	CA	120.83	17.46		119.18	21.00	
AVG N (μm)	AA	69.46	16.99	0.044^*^	68.15	15.50	0.13
	CA	77.09	17.84		73.52	14.39	
AVG S (μm)	AA	113.69	24.75	0.75	113.99	14.32	0.99
	CA	112.35	20.08		113.97	20.82	
AVG PMB (μm)	AA	44.04	10.75	0.10	47.28	8.31	0.67
	CA	47.31	10.73		46.78	10.74	
AVG TMV (mm^3^)	AA	8.17	0.51	0.003^*^	8.49	0.48	0.79
	CA	8.48	0.48		8.24	1.17	
AVG RNFL (mm^3^)	AA	0.74	0.14	0.013^*^	0.78	0.12	0.09
	CA	0.82	0.14		0.84	0.12	
AVG INL (mm^3^)	AA	0.98	0.07	0.36	0.97	0.05	0.29
	CA	0.97	0.53		0.95	0.69	
AVG OPL (mm^3^)	AA	0.77	0.04	0.050^*^	0.78	0.05	0.35
	CA	0.78	0.05		0.80	0.12	
AVG ONL (mm^3^)	AA	1.68	0.20	0.026^*^	1.59	0.19	0.000^*^
	CA	1.79	0.18		1.77	0.15	
AVG PR (mm^3^)	AA	2.16	0.30	0.51	2.22	0.05	0.43
	CA	2.22	0.05		2.17	0.28	
AVG RPE (mm^3^)	AA	0.52	0.91	0.39	0.38	0.02	0.74
	CA	0.36	0.03		0.40	0.25	
AVG GCIPL (mm^3^)	AA	1.79	0.258	0.038^*^	1.814	0.199	0.617
	CA	1.81	0.199		1.842	0.174	

*μm, micrometer; mm^3^, cubic millimeter; AA, African American; BMI, body mass index; CA, Caucasian American; T, temporal; I, inferior; S, superior; N, nasal; PMB, papillomacular bundle; TMV, total macular volume; RNFL, retinal nerve fiber layer; INL, internal nuclear layer; OCT, optical coherence tomography; OPL, outer plexiform layer; ONL, outer nuclear layer; PR, photoreceptor; RPE, retinal pigment epithelium; GCIPL, ganglion cell and internal plexiform layer*.

* *Statistically significant, multivariate analysis of covariance (MANCOVA)*.

### OCT in the Non-obese Group: AA vs. CA

There were non-statistically significant findings of the thickness of the pRNFL (G, T, I, N, S, or PMB) in AA vs. CA within the non-obese group. ONL macular volume was revealed to be significantly lower in AA vs. CA (*p* < 0.001) within the non-obese group. Non-significant difference noted of the macular layers (TMV, RNFL, INL, OPL, PR, RPE, or GCIP) between AA and CA within the non-obese group (Table 4).

### OCT Within the Same Race

Intra-racial analysis between obese and non-obese AA was significant for the T quadrant of pRNFL (*p* = 0.05). While there was a trend for significance for the RNFL and PMB (*p* = 0.06 and *p* = 0.07, respectively), differences for any other layers were non-significant. In CA, comparisons were non-significant for any retinal layer; however, there was a trend for significance for the INL (*p* = 0.07) (Table 5).

**Table 5:** Intra-racial analysis.

**OCT measure**	**BMI category**	**AA group**			**CA group**		
		**Mean**	**SD**	***p*-value**	**Mean**	**SD**	***p*-value**
AVG G (μm)	Obese	88.31	16.27	0.35	93.11	10.86	0.89
	Non-Obese	90.13	10.86		91.92	12.22	
AVG T (μm)	Obese	55.97	13.62	0.05^*^	62.35	16.80	0.69
	Non-Obese	60.47	9.99		62.63	13.47	
AVG I (μm)	Obese	114.47	21.77	0.22	120.83	17.45	0.88
	Non-Obese	118.39	16.82		119.18	21.00	
AVG N (μm)	Obese	69.46	16.99	0.98	77.09	17.84	0.40
	Non-Obese	68.15	15.50		73.52	14.38	
AVG S (μm)	Obese	113.69	24.75	0.74	112.32	20.08	0.59
	Non-Obese	114.00	14.32		113.97	20.81	
AVG PMB (μm)	Obese	44.04	10.75	0.07	47.31	10.73	0.99
	Non-Obese	47.28	8.31		46.78	10.73	
AVG TMV (mm^3^)	Obese	8.17	0.51	0.60	8.48	0.48	0.30
	Non-Obese	8.48	0.48		8.24	1.17	
AVG RNFL (mm^3^)	Obese	0.74	0.14	0.06	0.82	0.13	0.32
	Non-Obese	0.78	0.12		0.84	0.12	
AVG INL (mm^3^)	Obese	0.98	0.07	0.56	0.97	0.53	0.07
	Non-Obese	0.97	0.05		0.95	0.69	
AVG OPL (mm^3^)	Obese	0.77	0.05	0.47	0.78	0.05	0.45
	Non-Obese	0.78	0.05		0.80	0.12	
AVG ONL (mm^3^)	Obese	1.68	0.20	0.16	1.79	0.17	0.67
	Non-Obese	1.59	0.19		1.77	0.15	
AVG PR (mm^3^)	Obese	2.16	0.30	0.25	2.21	0.05	0.53
	Non-Obese	2.21	0.05		2.17	0.28	
AVG RPE (mm^3^)	Obese	0.52	0.90	0.42	0.36	0.03	0.27
	Non-Obese	0.38	0.02		0.40	0.24	
AVG GCIPL (mm^3^)	Obese	1.79	0.26	0.35	1.81	0.20	0.57
	Non-Obese	1.81	0.19		1.84	0.17	

*μm, micrometer; mm^3^, cubic millimeter; AA, African American; BMI, body mass index; CA, Caucasian American; T, temporal; I, inferior; S, superior; N, nasal; PMB, papillomacular bundle; TMV, total macular volume; RNFL, retinal nerve fiber layer; INL, internal nuclear layer; OPL, outer plexiform layer; OCT, optical coherence tomography; ONL, outer nuclear layer; PR, photoreceptor; RPE, retinal pigment epithelium; GCIPL, ganglion cell and internal plexiform layer*.

* *Statistically significant, multivariate analysis of co-variance (ANCOVA)*.

## Discussion

It is well-established that obesity has been associated with serious health risks, and its prevalence has been increasing, with almost 40% of the US population are now characterized as obese. Data from a National Health and Nutrition Examination Survey revealed that AA women are affected at a higher rate of 54.8% compared to non-AA women (38% for CA and 15% for Asian) (28, 29). Obesity is thought of as a chronic inflammatory state in which the adipocytes, under certain conditions, secrete pro-inflammatory cytokines, and downgrade anti-inflammatory cytokines. This leads to the attraction of leukocytes that continue to perpetuate a pro-inflammatory state by releasing more pro-inflammatory cytokines and producing reactive oxygen species (8, 30). Marrodan et al. reported a correlation between BMI and leptin levels, demonstrating increased proliferation of autoreactive T cells with high production of pro-inflammatory cytokines and inhibition of T regulatory cells (31). It is still controversial whether and to which extent the inflammatory component of obesity contributes to MS. However, given the chronic nature of MS, and the increased weight loss in its progressive stages, obesity may be a poor prognostic factor related to disease progression.

Our study showed a significant difference in the T quadrant of the pRNFL, INL, and the RNFL between the BMI groupings. Our results are in line with previous studies that have demonstrated the easy detectability and high frequency of the T quadrant of the pRNFL involvement in the MS population. After demonstrating a robust association between T quadrant atrophy and symbol digit modalities test (SDMT) and EDSS scores, Birkeldh et al. concluded that T quadrant atrophy is associated with increased cognitive and physical decline in patients with MS (32). Graham et al. reported significant thinning affecting the T quadrant in the non-ON eyes and also demonstrated that the T quadrant is a very specific marker for detecting changes over time (6). In another study on healthy young obese males, Dogan et al. demonstrated a negative association between BMI and T quadrant pRNFL thickness (33). Our findings support previous observations that the T quadrant is affected more than other pRNFL quadrants in our cohort of RRMS. Furthermore, we have shown that the T quadrant thickness is impacted more in patients with obesity than in patients with non-obesity, implying that this region might be more sensitive to the changes caused by obesity than other retinal structures.

Interestingly, in our intra-racial analysis, the T pRNFL was the only layer that reached significance in AA obese compared to AA non-obese, whereas non-significance was found in CA obese compared to CA non-obese. This finding raises some questions on whether the impact of obesity on T quadrant of pRNFL is race-driven or whether the AA patients with obesity are more obese with more vascular comorbidities affecting the retinal integrity compared with CA obese participants. Similarly, a trend for significance for the RNFL and PMB was found in AA obese, but not in obese CA compared with the non-obese counterparts.

We have also demonstrated the impact of elevated BMI on INL and RNFL. Previous studies have demonstrated that an increased INL volume has been linked to active inflammation. INL volume typically increases during clinical relapses and responds dynamically to treatment with disease-modifying agents (34, 35). Furthermore, it is correlated with gadolinium-enhancing lesions in brain MRI. In our study, INL volumes were significantly higher in the obese group compared with the non-obese. We assume that this finding might reflect the potential sensitivity of this layer to chronic low-grade inflammation associated with obesity; however, more research is needed to reach this conclusion. Although INL volume has not been associated with MS disease progression, our findings suggest that obesity-associated inflammation could potentially impact the integrity of this layer. In our intra-racial analysis, there was a trend for significance for the INL in CA obese compared with CA non-obese participants, whereas non-significance was reached in the intra-racial analysis in AA patients.

Furthermore, prior studies utilizing OCT have shown that inner layers, specifically the RNFL and GCIP, showed decreasing thickness with increasing EDSS score and decreasing brain volume, which may correlate more with worsening neurodegenerative and neuroinflammatory processes. (6, 34, 35). A number of studies reported that RNFL axonal loss is strongly correlated with changes in EDSS and clinical disability. Our findings reflect that following intra-retinal segmentation, the RNFL is impacted more significantly by obesity than the other layers, implying that the obesity-associated inflammatory process may also affect the neuroinflammatory and potentially neurodegenerative processes involved in MS disease progression. However, the difference in the GCIP layers did not reach significance, despite a trend for lower values in the obese group.

In the era of precision medicine and individualized treatment approach, we aim to investigate specific biomarkers that may help dictate treatment decisions. Therefore, we further investigate the effect of obesity on our AA and CA cohort. There is growing evidence that these two subgroups have different disease characteristics and AA patients, though not diagnosed with MS as frequently as CA patients, suffer from more aggressive disease course and increased disability (16, 17). AA experienced greater retinal injury compared with CA, regardless of history of ON (35, 36). Up to date, there is only one study that examined the effect of obesity on the retinal structures in the MS population. In a longitudinal observational study of 4.4 years, Filippatou et al. (37) have reported an accelerated rate of GCIPL atrophy in obese compared with normal weight patients with MS. Interestingly, no significance difference was found between overweight and normal weight participants (37).

In our race-based analysis, we observed that within the obese group, there are significant differences between the AA and CA patients in the following OCT measures: T, N, TMV, RNFL, OPL, ONL, and GCIPL. Prior studies demonstrated AA race was an independent factor associated with GCIPL atrophy, and our results confirmed previous observations (38). Furthermore, we demonstrated for the first time more severe injury of several other retinal layers in obese AA. Our findings suggest that obesity impacts retinal integrity in the AA population to a much greater extent than in the CA population, which would require more stringent monitoring of disease course for AA patients with obesity. Surprisingly, in the non-obese group, the ONL was the only layer found to be significantly thinner in AA compared to CA patients, which needs further investigation to determine whether it represents a biomarker for AA patients with MS or it only race-driven. The absence of normative data and the lack of a healthy control group do not allow any conclusions at this point; however, further research may shed light on the role of ONL layer in MS between races.

Our study has several strengths and weaknesses. We assume that our sample of 136 patients (272 eyes) overall is a decent sample size, even though no power calculations were performed. As a single center study, we use the same protocol, and the OCT assessments were performed in the same machine by the same technician. BMI documentation and OCT performance occurred at the same clinic visit. We applied strict inclusion and exclusion criteria, and we excluded patients with history of ON in either eye or other comorbidities; therefore, we minimized the impact of previous ON and other comorbidities on our results. However, given the lack of a healthy control group and the cross-sectional design, our study cannot prove casualty. We divided our patients into two groups: normal weight and obese, and we did not have a third “overweight” group. We use the BMI as a measure of body fat, despite body fat at a given BMI level can vary by sex, age, and race.

In summary, we demonstrated the impact of elevated body mass index on T quadrant of pRNFL, INL, and RNFL in our entire study cohort, and we reported a more severe impact of obesity in the AA population. The potential role of ONL as a race-specific biomarker is still unclear and needs further investigation. Thus, our findings should be considered preliminary but encouraging, and they should support efforts to further extend these observations to a larger cohort of patients with MS. Should our findings be confirmed on larger longitudinal studies, BMI monitoring, and maintenance of a healthy weight would have an important impact on the improvement of care of patients with MS.

## Data Availability Statement

The datasets presented in this article are not readily available because requests to access the datasets must first be approved by the Wayne State University Institutional Review Board. Requests to access the datasets should be directed to Evanthia Bernitsas, ebernits@med.wayne.edu.

## Ethics Statement

The studies involving human participants were reviewed and approved by Institutional Review Board, Detroit, MI. The patients/participants provided their written informed consent to participate in this study.

## Author Contributions

JR, MB, CB, and EB researched literature and conceived the study. MB, CB, FB, and MH were involved in data collection and analysis. JR and MB wrote the first draft of the manuscript. EB edited the final draft. All authors contributed to the article and approved the submitted version.

## Funding

This study was supported by the Sastry Foundation and Institutional support.

## Conflict of Interest

The authors declare that the research was conducted in the absence of any commercial or financial relationships that could be construed as a potential conflict of interest.

## Publisher's Note

All claims expressed in this article are solely those of the authors and do not necessarily represent those of their affiliated organizations, or those of the publisher, the editors and the reviewers. Any product that may be evaluated in this article, or claim that may be made by its manufacturer, is not guaranteed or endorsed by the publisher.
